# Current Insight into Traditional and Modern Methods in Fungal Diversity Estimates

**DOI:** 10.3390/jof8030226

**Published:** 2022-02-24

**Authors:** Ajay Kumar Gautam, Rajnish Kumar Verma, Shubhi Avasthi, Sushma, Yogita Bohra, Bandarupalli Devadatha, Mekala Niranjan, Nakarin Suwannarach

**Affiliations:** 1 School of Agriculture, Abhilashi University, Mandi 175028, Himachal Pradesh, India; 2 Department of Plant Pathology, Punjab Agricultural University, Ludhiana 141004, Punjab, India; yogitabohra@pau.edu; 3 School of Studies in Botany, Jiwaji University, Gwalior 474011, Madhya Pradesh, India; shubh.avasth@gmail.com; 4 Department of Biosciences, Chandigarh University, Gharuan 140413, Punjab, India; smunihal@gmail.com; 5 Fungal Biotechnology Lab, Department of Biotechnology, School of Life Sciences, Pondicherry University, Kalapet 605014, Pondicherry, India; devadatha796@gmail.com; 6 Department of Botany, Rajiv Gandhi University, Rono Hills, Doimukh, Itanagar 791112, Arunachal Pradesh, India; neeru436@gmail.com; 7 Research Center of Microbial Diversity and Sustainable Utilization, Chiang Mai University, Chiang Mai 50200, Thailand

**Keywords:** classical and molecular methods, fungal diversity, fungal phylogeny, identification, taxonomy

## Abstract

Fungi are an important and diverse component in various ecosystems. The methods to identify different fungi are an important step in any mycological study. Classical methods of fungal identification, which rely mainly on morphological characteristics and modern use of DNA based molecular techniques, have proven to be very helpful to explore their taxonomic identity. In the present compilation, we provide detailed information on estimates of fungi provided by different mycologistsover time. Along with this, a comprehensive analysis of the importance of classical and molecular methods is also presented. In orderto understand the utility of genus and species specific markers in fungal identification, a polyphasic approach to investigate various fungi is also presented in this paper. An account of the study of various fungi based on culture-based and cultureindependent methods is also provided here to understand the development and significance of both approaches. The available information on classical and modern methods compiled in this study revealed that the DNA based molecular studies are still scant, and more studies are required to achieve the accurate estimation of fungi present on earth.

## 1. Introduction

Biodiversity is one of the most interesting aspects of biology, which has attracted the attention of scientists and researchers for some time. Biological diversity generally represents the variety of living beings from all sources, including terrestrial, marine and other aquatic ecosystems, covering the diversity of plants, animals, insects, pests and microbes. The information on biodiversity yet to be fully discovered may be useful from many beneficial and harmful aspects of life. Based on available information, biodiversity can be of species which are genetic and ecological, and found to be distributed in a variety of environments. The various life forms are adapted to live in specific environments, referred to as terrestrial and aquatic. In addition, these diverse life forms show great variability based on the type of habitats [1]. Fungi is an important component of biodiversity, which play an important role in various ecological cycles [2,3].

Fungi present enormous species diversity with respect to morphological, ecological and nutritional modes. Fungi are considered the largest organismic group after insects [4], andareknown to exist in a wide variety of morphologies, lifestyles, developmental patterns anda wide range of habitats such as soil, water, air, animals, plants and in environments with extreme conditions such as low or high temperature, high concentration of metals and salts [5,6,7]. It has been estimated that 1.5 and 5.1 million species of fungi are believed to exist in various ecosystems of Earth, of which nearly 150,000 species of fungi have been described [8,9,10].

Fungi are an important and diverse component of biodiversity in various ecosystems. These organisms consist of a diverse range of all major fungal groups and play the role of both foe and friend. While some fungi may cause numerous diseases in humans, animals, plants and other biological substrates, others may play an important role in the nutrient cycle. In addition, fungi have beneficial applications in the agriculture, industrial and pharmaceutical sectors. The occurrence of fungi, however, varies greatly with respect to various ecosystems and environments. The study of fungi is not easy due to the extremely high level of diversity and difficulty in the prediction ofexact estimates. However, different researchers predicted fungal diversity on the planet and provided different estimates of fungal species [3,11,12].

Identification based on morphological, phylogenetic or ecological characteristics is one of the most important aspectsof mycological studies. The classical methods of fungal identification which rely on direct observation of fungi either in a natural condition or after culturing on growth media are still most popularly in use. Despitethe use of molecular methods as more advanced modern techniques of fungal identification, the classical methods still have many advantages for studying fungal diversity. Some fungi produce visible structures useful in their identification. The culturing of some of the fungi is still not very successful; therefore, molecular techniques have proved to be very helpful in exploring their taxonomic identity [13,14]. The use of molecular methods along with conventional methods (morphological studies) helped mycologists to investigate the new fungal samples or reinvestigate the preserved ones. This has been led the fungal taxonomists to propose or establish many new taxa.

In the present paper, a general outline with current estimates of fungal diversity in all environments is presented. A complete section on general methods (classical and modern methods) used for fungal identification, along with their advantages and disadvantages, was also presentedin order to provide an updated account on fungal identification. Moreover, adetailed account of culture dependent and culture independent methods was providedin order to highlight their importancein fungal identification and their usefulness in finding updated fungal diversity estimates. Overall, this review will be a document containing present day information on various aspects of fungi.

## 2. Fungal Diversity: General Outline with Updated Estimates

Fungi constitute one of the largest groups of eukaryotes which play a significant role as decomposers, mutualists and pathogens. They are among the key components of global biodiversity, playing a powerful role in global biogeochemistry, recycling carbon and mobilizing nitrogen, phosphorus and other bio-elements. Besides performing this key role, fungi provide essential support to plant life in the form of endophytes and mycorrhizae, in addition to causing numerous plant and animal diseases. The industrial applications of various fungi nowadays are worth appreciating. Fungi as an important food source, and researchis still in progress to use fungal biomass to fulfil the basic needs of food, clothe and shelter [15,16]. Despite multiple uses, updated information of these organisms about the number of species are described, as well as global estimates of their diversitywhich are essential to accurately describe their taxonomic characteristics. Through the use of advanced methods of isolating and identifying fungi, a number of novel taxa have been established over the past decade, including new divisions, classes, orders and new families. Therefore, this section provides complete information on how to estimate fungal diversity based on the available literature.

**Figure 1:**
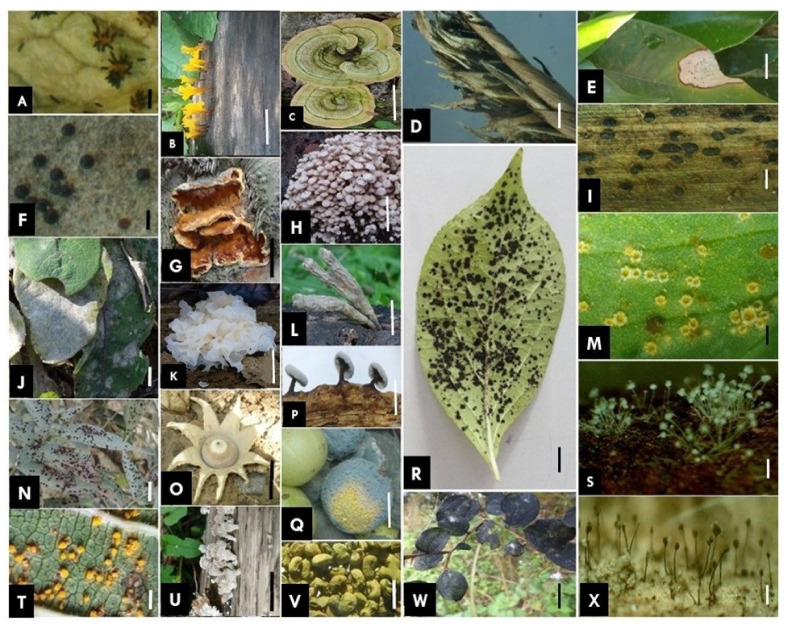
Diversity of different types of fungi. (**A**) *Phragmidium* sp. [rose rust], (**B**) *Calocera* sp., (**C**) *Trametes* sp., (**D**) *Tilletia* sp. [smut], (**E**) *Colletotrichum* sp. [Leaf spot], (**F**) *Erysiphe* sp. [Powdery mildew cleistothecia], (**G**) *Inonotus* sp., (**H**) *Termitomyces* sp., (**I**) *Kweilingia* sp. [rust], (**J**) *Podosphaera* sp. on *Sonchus* sp. [Powdery mildew], (**K**) *Tremella* sp., (**L**) *Xylaria* sp., (**M**) *Uromyces* sp. [aecia and telia], (**N**) *Pileolaria* sp. [rust], (**O**) *Gaestrum* sp., (**P**) *Didymium* sp., (**Q**) *Penicillium* sp. on *Emblica* sp., (**R**) *Schiffnerula* sp. [black mildew], (**S**) *Aspergillus* sp., (**T**) *Coleosporium* sp. [rust], (**U**) *Schizophyllum* sp., (**V**) *Aspergillus* sp. [on cow pea], (**W**) *Mitteriella* sp. [black mildew] and (**X**) *Periconia* sp. Scale bars A–X = 20 mm.

**Figure 2:**
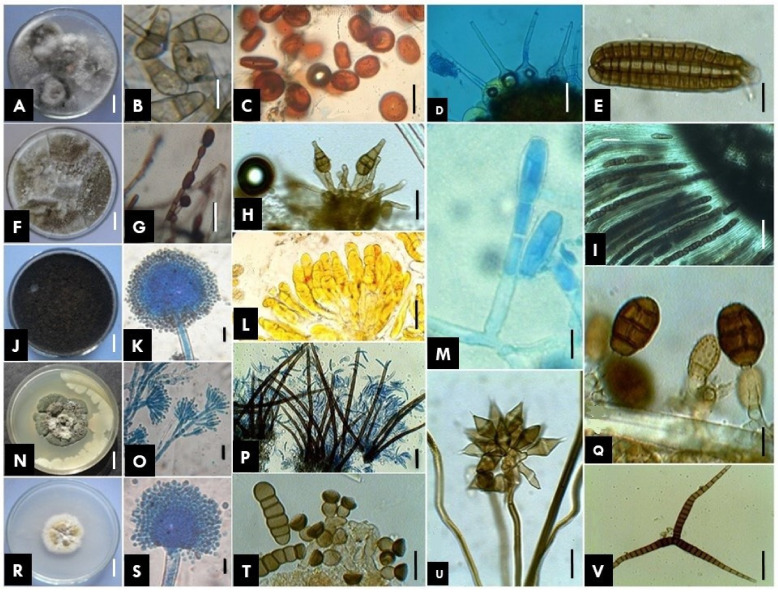
Diversity of different types of fungi. (**A**,**B**) *Curvularia* sp., (**C**) *Pileolaria* sp. [rust], (**D**) *Phyllactinia* sp. [powdery mildew], (**E**) *Dictyosporium* sp., (**F**,**G**) *Sytalidium* sp., (**H**) *Alternaria alternata*, (**I**) *Hypoxylon* sp., (**J**,**K**) *Aspergillus niger*, (**L**) *Coleosporium* sp. [rust], (**M**) *Podosphaera* sp. [powdery mildew], (**N**,**O**) *Penicillium* sp. on *Emblica* sp., (**P**) *Colletotrichum* sp., (**Q**) *Pithomyces* sp., (**R**,**S**) *Aspergillus falvus*, (**T**) *Torula* sp., (**U**) *Beltrania* sp., (**V**) *Ceratosporium* sp. Scale bars A,F,J,N,R = 1 mm; B–E,G–I,K–M,O–Q,S–V = 10 µm.

Classification of fungi or their various groups is a continuous process because of the regular inclusion of data based on morpho-taxonomy and molecular studies. The frequent inclusion of data from DNA sequences in recent studies is updating fungal outlines and their estimates constantly. The outline of fungi classification provided by Wijayawardene et al. [17] is used here as a starting point for this section of the paper. An outline of fungi and fungus-like taxa provides a summary of the classification of the kingdom Fungi (including fossil fungi, i.e., dispersed spores, mycelia, sporophores and mycorrhizas). A total of 19 phyla were presented with the placement of all fungal genera with the described number of species per genus at the class-, order-and family-level [17]. Several earlier studies have also focused on fungal diversity. Some glimpses of different types of fungi found in various habitats are presented in Figure 1 and Figure 2.

Based on phylogenies and the divergence time of particular taxa, Tedersoo et al. [18] proposed classification of kingdom Fungi into 18 phyla Ascomycota, Aphelidiomycota, Basidiobolomycota, Basidiomycota, Blastocladiomycota, Calcarisporiellomycota, Chytridiomycota, Entomophthoromycota, Entorrhizomycota, Glomeromycota, Kickxellomycota, Monoblepharomycota, Mortierellomycota, Mucoromycota, Neocallimastigomycota, Olpidiomycota, Rozellomycota and Zoopagomycota. Because this study was based on only 111 taxa, its universal acceptance remained a matter of thinking. In this agreement, Wijayawardene et al. [19] provided a revised classification system for basal clades of fungi from phyla to genera in the same year. A total of 16 phyla were accepted among the above-mentioned except *viz.* Ascomycota and Basidiomycota. The detailed information to fully resolved tree of life was reviewed by James et al. [20], where they provide detailed information on advancements in genomic technologies during the last 15 years to understand the revolution in fungal systematics in the phylogenomic era. However, the recently updated outline of fungi given by Wijayawardene et al. [17] revised the number of phyla upto 19 in addition to Caulochytriomycota. This group of researchers also included fungal-like taxa in this study and incorporated them in this outline. Similar studies on outlined fungal phyla were carried outoccasionally. These studies proved very useful for researchers engaged in updating fungal classification. A list of selected literature based on various taxonomical studies carried out by several researchers is presented in Table 1.

These are studies on defining boundaries and providing the classification of different levels of fungal classification: Ascomycota [21,22,23], Diaporthales [24,25,26,27,28,29], Leotiomycetes [30], Magnaporthales [31], Orbiliaceae (Orbiliomycetes) [32], Discomycetes [33], Sordariomycetes [34,35,36], Sclerococcomycetidae [35,37], Xylariales [38], Xylariomycetidae [39] and Pezizomycetes [40]. Based on this, a brief outline of the classification of the kingdom Fungi (including fossil fungi, i.e., dispersed spores, mycelia, sporophores, mycorrhizas) given by Wijayawardene et al. [17] is provided herein tabulated form (Table 2).

**Table 1:** Selected literature on various taxonomical studies of fungi.

Title	Reference
Orders of Ascomycetes	[41]
Laboulbeniales as a separate class of Ascomycota, Laboulbeniomycetes	[42]
One hundred and seventeen clades of euagarics	[43]
Toward resolving family-level relationships in rust fungi (Uredinales)	[44]
Higher level classification of Pucciniomycotina based on combined analyses of nuclear large and small subunit rDNA sequences	[45]
A phylogenetic overview of the family Pyronemataceae (Ascomycota, Pezizales)	[46]
A higher-level phylogenetic classification of the Fungi	[47]
Dictionary of the Fungi. (10th edn)	[48]
Outline of Ascomycota	[49]
*Glomeromycota*: two new classes and a new order	[50]
*Entomophthoromycota*: a new phylum and reclassification for entomophthoroid fungi	[51]
Incorporating anamorphic fungi in a natural classification checklist and notes for 2011	[52]
Taxonomic revision of *Ustilago*, *Sporisorium* and *Macalpinomyces*	[53]
Phylogenetic systematics of the Gigasporales	[54]
List of generic names of fungi for protection under the International Code of Nomenclature for algae, fungi, and plants	[55]
A phylogeny of the highly diverse cup fungus family Pyronemataceae (Pezizomycetes, Ascomycota)	[56]
Families of Dothideomycetes	[57]
Taxonomic revision of the Lyophyllaceae (Basidiomycota, Agaricales) based on a multigene phylogeny	[58]
Recommended names for pleomorphic genera in Dothideomycetes	[27]
Towards a natural classification and backbone tree for Sordariomycetes	[34]
Phylogenetic classification of yeasts and related taxa within Pucciniomycotina	[59]
Entomophthoromycota: a new overview of some of the oldest terrestrial fungi	[60]
Systematics of Kickxellomycotina, Mortierellomycotina, Mucoromycotina, and Zoopagomycotina	[61]
A phylum-level phylogenetic classification of Zygomycete fungi based on genome–scale data	[62]
Phylogenomics of a new fungal phylum reveals multiple waves of reductive evolution across Holomycota	[63]
Sequence–based classification and identification of fungi	[64]
Morphology-based taxonomic delusions: *Acrocordiella*, *Basiseptospora*, *Blogiascospora*, *Clypeosphaeria*, *Hymenopleella*, *Lepteutypa*, *Pseudapiospora*, *Requienella*, *Seiridium* and *Strickeria*	[65]
Families of Sordariomycetes	[35]
Proposal to conserve the name Diaporthe eres, with a conserved type, against all other competing names (Ascomycota, Diaporthales, Diaporthaceae)	[66]
Taxonomy and phylogeny of dematiaceous Coelomycetes	[67]
Multigene phylogeny of Endogonales	[68]
Classification of lichenized fungi in the Ascomycota and Basidiomycota-Approaching one thousand genera	[69]
Taxonomy and phylogeny of the Auriculariales (Agaricomycetes, Basidiomycota) with stereoid basidiocarps	[70]
An updated phylogeny of Sordariomycetes based on phylogenetic and molecular clock evidence	[71]
Families, genera, and species of Botryosphaeriales	[72]
Ranking higher taxa using divergence times: a case study in Dothideomycetes	[73]
A revised family-level classification of the Polyporales (Basidiomycota)	[74]
Notes for genera: Ascomycota	[22]
Towards incorporating asexual fungi in a natural classification: checklist and notes 2012–2016	[23]
Notes for genera: basal clades of Fungi (including Aphelidiomycota, Basidiobolomycota, Blastocladiomycota, Calcarisporiellomycota, Caulochytriomycota, Chytridiomycota, Entomophthoromycota, Glomeromycota, Kickxellomycota, Monoblepharomycota, Mortierellomycota, Mucoromycota, Neocallimastigomycota, Olpidiomycota, Rozellomycota and Zoopagomycota)	[19]
Outline of Ascomycota: 2017	[75]
Classification of orders and families in the two major subclasses of Lecanoromycetes (Ascomycota) based on a temporal approach	[76]
A taxonomic summary and revision of *Rozella* (Cryptomycota)	[77]
Sexual and asexual generic names in Pucciniomycotina and Ustilaginomycotina (Basidiomycota)	[78]
Evolutionary complexity between rust fungi (Pucciniales) and their plant hosts	[79]
High-level classification of the Fungi and a tool for evolutionary ecological analyses	[18]
Taxonomy and phylogeny of operculate Discomycetes: Pezizomycetes	[33]
Molecular phylogeny of the Laboulbeniomycetes (Ascomycota)	[80]
Families in Botryosphaeriales	[81]
Natural classification and backbone tree for Graphostromataceae, Hypoxylaceae, Lopadostomataceae and Xylariaceae	[82]
Classification of the *Dictyostelids*	[83]
Revisiting Salisapiliaceae	[84]
Phylogenetic revision of Savoryellaceae	[85]
Notes, outline and divergence times of Basidiomycota	[86]
A new phylogenetic classification for the Leotiomycetes	[87]
Taxonomy and phylogeny of hyaline-spored Coelomycetes	[88]
Refined families of Sordariomycetes	[36]
Outline of Fungi and fungus-like taxa	[17]
The genera of Coelomycetes	[89]
A higher-rank classification for rust fungi, with notes on genera	[90]
Indian Pucciniales: taxonomic outline with important descriptive notes	[91]
Incorporating asexually reproducing fungi in the natural classification and notes for pleomorphic genera	[92]
How to publish a new fungal species, or name, version 3.0	[93]

**Table 2:** A brief presentation on outline of fungi.

Phylum	Class	Order	Family	Genera
Aphelidiomycota	1	1	1	4
Ascomycota	21	148	624	4511
Basidiobolomycota	1	1	1	2
Basidiomycota	19	69	240	1521
Blastocladiomycota	2	4	8	12
Calcarisporiellomycota	1	1	1	2
Caulochytriomycota	1	1	1	1
Chytridiomycota	9	13	52	97
Entomophthoromycota	2	2	5	20
Entorrhizomycota	1	2	2	2
Glomeromycota	3	5	16	49
Kickxellomycota	6	6	7	61
Monoblepharomycota	3	3	7	9
Mortierellomycota	1	1	1	6
Mucoromycota	3	3	17	62
Neocallimastigomycota	1	1	1	11
Olpidiomycota	1	1	1	4
Rozellomycota	2	7	41	162
Zoopagomycota	1	1	5	25
**Total**	**79**	**270**	**1031**	**6561**

**Figure 3:**
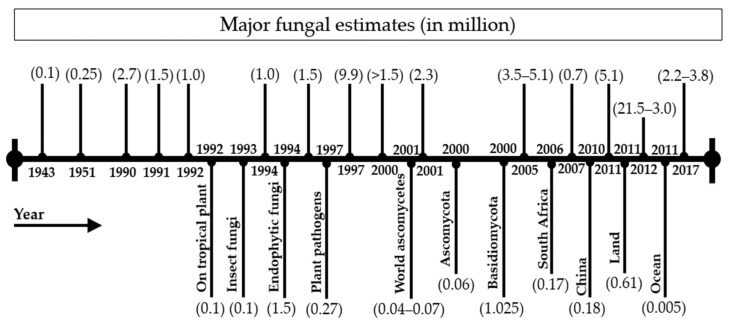
Estimations on the global number of fungal species.

As one of the ancient and most diverse branches of the tree of life, kingdom Fungi contains an estimated 4–5 million species distributed all across the globe and plays vital roles in terrestrial and aquatic ecosystems [94,95,96,97]. Of the total estimated number, so far, less than 2% of fungiis described [98]. Because of the vast diversity of these organisms and the addition of new fungi year by year, mycologists are facing major difficulties to define their boundaries accurately. The regular advancement in mycological techniques enables mycologists to describe new fungi all around the world every year based on decade evaluations. The description and addition of new species are estimated at 2626 from 2000 to 2012, while it was around 2326 between 1980 and 1999 [99,100,101]. This ongoing process of describing new fungi changes the overall estimate of fungi regularly. However, the suspense of undescribed fungi is still the same, which also added more uncertainty over defining their estimate exactly. In addition to natural habitats still waiting to explored, requirements of reassessment of dried herbarium samples based on molecular methods, along with morpho-taxonomy and lack of molecular facilities, still hinder mycologists in describing new fungi and attaining full estimate boundaries. Because of the importance of a total number of fungi estimates in their diversity and taxonomy (systematics, resources and classification) [12,102], many estimates have been put forward to elucidate the fungal species diversity in the world. Previous estimates of fungal diversity were based mainly on the plant-associated fungi [3]. Summarizing a comprehensive account of previous estimates of fungal diversity, we start with the estimate of about 100,000 presented by Bisby and Ainsworth [102]. Then, the number of fungi was estimated to be between 0.25–2.7 during the second half of the twentieth century. It was estimated (in millions) as follows: 0.25 [103], 2.7 [104], 1.5 [12,105,106], 1.0 [107,108,109], 1.3 [110], 0.27 [111] and 0.5 [112]. Similarly, the estimates on total described numbers of fungi during the twenty-first century were found to be between 2.3–5.1 million. The fungal estimate (1.5 million) provided by Hawksworth [12] has been most widely accepted for two decades. However, updated estimates of fungal species were provided in the current century as 3.5–5.1 [113], 5.1 [10], 2.2–3.8 [11]. The updated estimates were provided based on DNA based molecular techniques and next-generation sequencing. However, Hyde et al. [114] pointed out that more than 90% of the collected samples of fungi were neglected by mycological taxonomists around the globe. The total number of described fungi may be increased many times after processing these samples. The fungal estimates provided by various mycologists are presented in detail in Figure 3.

In addition to estimating the total number of fungi, the global biodiversity of fungi has been extensively investigated for predicting their accurate estimate on earth. The number of advanced techniques, along with the number of numerical analytical methods, enabled researchers not only to identify and describe those fungi which are either not described, incorrectlyidentified or described incompletely, but also in understanding plant: fungus ratios [12,99], quantitative macroecological grid-based approaches [115,116,117], ecological scaling laws and methods based on environmental sequence data including plant: fungus ratios [10,113]. These studies on estimates proved fungi to be one of the largest groups of living organisms on this planet. An updated estimate of global fungal diversity is 2.2 to 3.8 million provided by Hawksworth and Lücking [11], however, also pointed out that this estimate would be a thousand times higher than the current highest estimate of 10 million species. A regression relationship between time and described fungal species by using Sigma State 3.5.SPSS (USA) was constructed and presented by Wu et al. [3]. With the help of this equation model, Wu et al. [3] presented the description rate of fungi. They indicated that 1.5 million fungal species, estimated by Hawksworth [12], could be described only by the year 2184, while the estimates of 2.2 and 3.8 million could be described by the years 2210 and 2245, respectively.

## 3. General Methods of Fungal Identification

The correct identification of fungi is one of the essential tools required for documenting fungi at the genus and species levels. There are several methods of fungal identification that differ in scope and content. However, the actual identification procedure is almost the same in each of the methods. Colonial morphological features, along with growth rate and microscopic observations, are some important criteria used to study different fungi. However, technological advancements have added more improved and sophisticated methods in this series. Generally, the fungal identification techniques are, broadly, three types, i.e., truly classical, culture and modern methods. While truly classical methods were based on the study of morphological features, the culture methods involved culture media technique. In modern methods, DNA-based techniques are utilized.

### 3.1. Classical Methods

Classical methods are most widely used in the documentation of fungi in relation to their identification and distribution on any substrate over a specific area. In general, these methods have been developed for studying any substratum or group of fungi [118]. Classical methods of fungal identification generally include incubation of substrata in moist chambers, direct sampling of fungal fruiting bodies, culturing of endophytes and particle plating. The following are basic types of classical methods.

#### 3.1.1. Opportunistic Approach

In general, the opportunistic approach is one of the different types of classical methods used by mycologists to collect fruiting bodies of macromycetes. The availability of good condition fruiting bodies of macrofungi is generally a prerequisite for this efficient method of detecting new species or new records in a study area. The requirement of highly skilled mycologists for collection, processing and identification is a major limitation of this method, along with the risk of toxicity from these fungi [118].

#### 3.1.2. Substrate Based Approach

The substrate-based protocols are another important approach used for the identification of fungi. The importance of these methods can be imagined because while some fungi fruit rather dependably, others fruit only sporadically. The substrate-based methods are mostly used for fungi that occur only on discrete, discontinuous or patchy resources, or are restricted to a particular host. The fungi forming sporocarps on soil, trees, large woody stumps, leaf litter, twigs and small branches are generally included in such methods. The fungi that form fruiting bodies on soil and ectomycorrhizal association with the trees provides a better understanding of their identification and diversity. The selection of a study plot is an important step that should be considered while using these methods [119,120]. In the case of fungi that form fruiting bodies on large woody debris, use of the log-based sampling method is generally preferred, keeping in view the substrate characteristics such as diameter, decay classes, upright, suspended, or grounded and host information [118,121]. Similarly, the use of a plot-based or band transect method is generally suggested in fungi, giving rise to fruiting bodies on fine debris (leaf litter, twigsand small branches). Here, size of the sample plot is generally kept in mind during the collection of fungal samples [119,120,122,123,124].

#### 3.1.3. Moist Chambers Techniques

Moist Chambers Techniquesis one of the earliest and more effective methodsbeing utilized by mycologists in fungal taxonomy. This technique is used for fungi growing on leaves or small woody debris, such as ascomycetes, hyphomycetes and coelomycetes [124,125] and slime molds [126], and fungi growing on dung [127,128,129,130]. Here, the fungal samples collected from various substrates were processed for the production of fruiting bodies in a moist chamber for some duration and evaluated periodically for approximately 2 to 6 weeks.

#### 3.1.4. Culture Media Technique

The use of culture media to inoculate fungi from the natural environment and incubate it to grow in controlled conditions for their isolation and identification is also one of the popular and widely used techniques. Numbers of artificial culture media are used here to provide growth substrate and required nutrition to inoculated fungi. Along with morphological characteristics, this technique proves quite useful in identifyinga fungal taxon. The easy and economic implication of this method has made it popular among mycologists. The numberof fungal groups such as endophytes, saprophytes and parasites—except obligate—can be isolated on various culture media from symptomless but fully expanded leaves, petioles, twigs, branches and roots, etc. [131]. Similarly, culturing of leaf washes is another culture media-based technique to assess the composition of spores on leaf surfaces. Commonly known as phylloplane fungi, these are considered to have good biocontrol potential [132,133]. Another culture based method known as the particle filtration method [134,135,136] is mainly meant for reducing the number of isolates derived from dormant spores in cultures taken from decomposing plant debris. Vegetatively active mycelia are generally cultured with the use of this method.

#### 3.1.5. Advantages and Disadvantages of Truly Classical and Culture Based Methods

When we compare classical and culture based-methods with other advanced techniques, they still hold a key position in all the methods being utilized for assessing identification, diversity and distribution of fungi. Although these techniques are still in use globally, they also have certain disadvantages. In order tomake mycologistsaware of all aspects of basic methods (truly classical and culture based), a brief discussion on some of their important advantages/disadvantages is given below:

##### Advantages of Truly Classical and Culture Based Methods

- These methods are still considered as the sources which can provide complete information on fungal communities of different areas with variable habitats. Because of the non-availability of DNA-based sequence data of all the fungi, it is the only criteria to determine basic information about individual species, such as geographic range, host relationships and ecological distribution.
- The effects of abiotic variables (pH, soil nutrient content, weather-related variables) and biotic variables on fungi of the variable substrate and environmental conditions can be more easily studied by these methods.
- As compared to an advanced one, these methods are more economical and can be executed with less specialized equipment.
- Overall, the developing nations where adequate research funding is still a big challenge; these methods are important considerations for many investigators.

##### Disadvantages of Classical and Culture Based Methods

- For the fungi which are unable to grow or produce reproductive structures on culture or hardly reproduce naturally, these methods are not suitable and become a major limitation in identifying, classifying and outlining fungi of a specific area.
- The detailed procedure of sampling, culturing, isolation and identification methods are considerably more time consuming in comparison to more advanced techniques. The confirmation of new genera or species can be predicted more efficiently and accurately from the repeatedly sampled areas [120].
- Due to the above-mentioned disadvantages, classical taxonomists are now considered to be endangered, as the interests of young researchers in classical methods is considerably reducing. If one willing to peruse a career in classical mycology, it takes a long duration of training. Similarly, to identify all of the collections based on the classical approach increases the time duration to find out final results. In molecular methods, technical expertise is quite enough to carry out research which also poses a major limitation to classical methods.

#### 3.1.6. Advantages and Disadvantages of DNA Based Modern Methods

Besides having many advantages, the DNA-based methods also have some limitations, while modern methods are proven to be more efficient in the confirmation of new genera or species inlesser in time consumption. When classical methods are not able to study the fungi more specifically due to overlapping characters, i.e., a high degree of phenotypic plasticity, cryptic species and occurrence of different morphs for the same taxa [67,137,138], there are molecular methods which prove helpful to resolve such issues more accurately.

Like other methods, these methods also have certain disadvantages. The information we obtained with the help of this method is not so detailed as to be compared to classical methods; e.g., when we study basidiomata classically, we obtain a lot of information that we will never learn from DNA. Based on DNA-based techniques, numbers of new species are proposed, solely on the basis of unavailability of their sequences in the databases. Additionally, the submission of improper DNA sequences of many described fungi without proper editing is another drawback caused by molecular methods. Besides, poor taxon coverage in public depositories remains the principal impediment for successful species identification through molecular methods. The interpretation of BLAST results is regarded as the most important aspect in DNA-based methods of fungal identification. The availability of appropriate taxonomic and molecular experts in limited numbers is one of the major drawbacks of these methods. In addition, the contamination of DNA samples is another problem associated with molecular methods. Lastly, these methods are not cost effective in comparison to classical ones.

Keeping in view both advantages and disadvantages, it was found that mycological studies based on classical methods can perform better when combined with molecular analyses.

## 4. Assessment of Fungal Taxonomy and Diversity

Fungal taxonomy is the fundamental aspect of immense value utilized during mycological studies. The taxonomy of fungi based on morphological characters has been used for centuries and is still in use. Fungal taxonomy is generally required to identify and define existing and new fungi, andis ultimately useful in the assessment of their diversity and distribution. With the passage of time, the use of new and varied methods of fungal assessment came into existence which revolutionize the traditional methods based on morpho-taxonomy. However, both the methods based on morphology and molecular data care are still used equally and have their own levels of importance. It is primarily significant to use morphological-based methods and follow other approaches such as chemical, ecological, molecular or physiological analyses [139]. However, some technologies are expensive or inconvenient in terms ofuse in laboratories where the infrastructure is basic. Morphological analyses are, however, low-cost and results are acquired rapidly. These novel technologies have a relatively high cost. In cases wherethere is a limited quantity of a specimen or lack of sequence data, morphological data then play an important role in identification. In GenBank, there are many sequences which are wrongly named with errors. In such cases, detailed and extensive morphological characters help to resolve the taxonomy of them [140]. Therefore, morphology is still the most common technique to study fungi.

However, in recent times progress has driven taxonomic inferences towards DNA-based methods, and these procedures have parallel pros and cons. Modern mycotaxonomy has moved onward using morphological characters with a combination of chemotaxonomy, ecology, genetics, molecular biology and phylogeny [139,141,142,143,144,145]. The exploitation of sequence data for phylogenetic, biological, genetic and evolutionary analyses has offered a lot of understanding into the diversity and relationships of various fungal groups [71,139,146,147,148].

In DNA-based molecular characters, culture dependent and culture-independent methods are in practice nowadays to estimate fungal diversity. Culture-based approaches have been traditional, used to analyse microorganisms in indoor environments, including settled floor dust samples. However, this approach can be biased, for example, by microbial viability and/or culturability on a given nutrient medium. The advent of growth-independent molecular biology-based techniques, such as polymerase chain reaction (PCR) and DNA sequencing, has circumvented these difficulties. However, few studies have directly compared culture-based morphological identification methods with culture-independent DNA sequencing-based approaches. For example, a previous study compared the presence or absence of fungal species detected by a culture-based morphological identification method and a culture independent DNA sequencing method [149]. However, only a qualitative comparison was conducted between these two different approaches and a quantitative comparison was not conducted (Table 3 and Table 4). A detailed account of general tools and repositories generally used in DNA-based identification of fungi are presented in Table 5.

**Figure 4:**
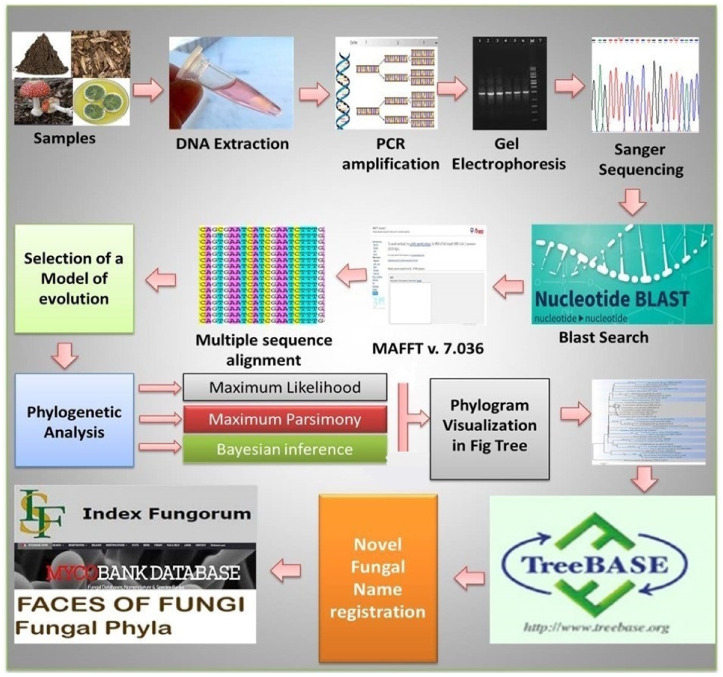
An overview of DNA-based molecular techniques used in fungal sample analyses.

**Figure 5:**
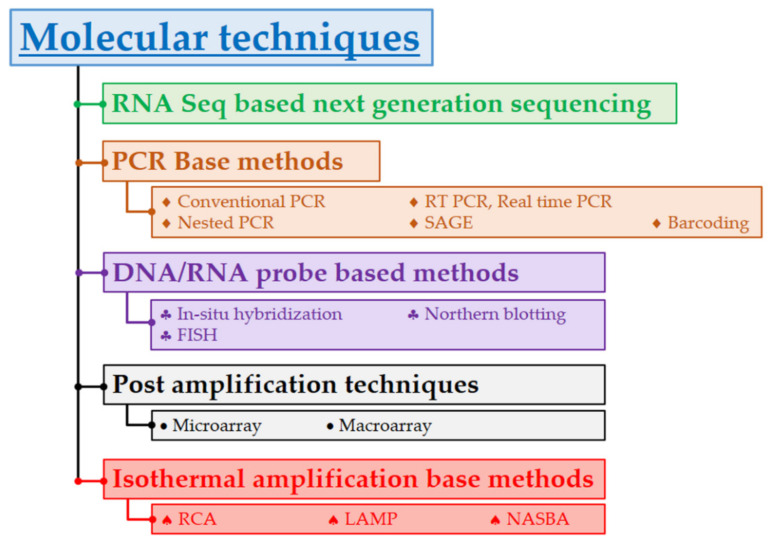
Different molecular techniques used in DNA-based analyses of different fungi.

Likewise, a listing of Sequence Independent methodsand High-throughput sequencing platforms are summarized in Table 6. The pictorial overview on different molecular techniques, as well as the general protocol of culture dependent and culture independent DNA-based molecular techniques used in fungal sample analyses, is also present here (Figure 4 and Figure 5).

## 5. Polyphasic Identification

The correct identification of species is a crucial goal in taxonomy. Information about each identified fungal species (e.g., biochemical properties, ecological roles, morphological description, physiological and societal risks or benefits) is a vital component in this process. Identification is a never-ending and apparently lengthy process with several amendments of the taxonomic outlines. 

**Figure 6:**
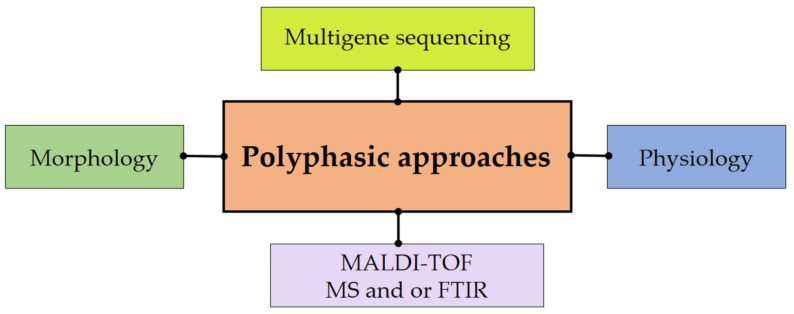
Modern polyphasic methodology of fungal identification.

The polyphasic approaches comprise the use of varied procedures based on the grouping of scientific information. Various approaches such as biochemical, micro-and macro-morphology, and molecular biology studies are applied (Figure 6). Microbial spectral analysis based on mass spectrometry (particularly matrix assisted laser desorption/ionization time-of-flight mass spectrometry//MALDI-TOF MS) has been developed and used as an important step in the polyphasic identification of fungi [355].

A polyphasic method based on ecology, morphology and molecular data based techniques (multigene sequencing) is highly advocated to identify the fungal species precisely. Phylogenetic analyses have been comprehensively used to interpret species limitations in several fungal genera [356,357] shown in Table 7. There are several fungal species that have not been correctly identified. However, there are numerous boundaries associated with phylogenetic analyses for species identification [358,359]. There is an absence of molecular data for many fungal species, including reference sequences, and few species only have ITS sequences, which obstructs molecular-based techniques [360,361]. Moreover, phylogenetic analyses do not account for hybridization events and horizontal gene transfer [359]. The internal transcribed spacer (ITS) region has been accepted as a nearly universal barcode for fungi owing to the ease of amplification and its wide utility across the kingdom; however, it can often only be used for placement of taxa up to the genus level [361,362]. There is also a lack of ex-type or authenticated sequences for several pathogenic genera [355]. The identification of species boundaries is, thus, important to better understand genetic variation in nature to develop sustainable control measures [363].

It is also recommended to use diverse methods, including Bayesian inference, maximum likelihood, maximum parsimony coupled with automatic barcode gap discovery, coalescent-based methods or genealogical concordance phylogenetic species recognition to explore species boundaries in various fungal genera [358,360,364].

**Table 7:** An overview of polyphasic approach on analyses of plant pathogenic fungi.

Family	Genus	Genetic Marker for Genus Level	Genetic Markers for Species Level	References
Pleosporaceae	*Alternaria*	LSU and SSU	ITS, *GAPDH*, *rpb2* and *tef1-α*	[365,366,367,368]
Physalacriaceae	*Armillaria*	ITS	ITS, *IGS1* and *tef1-α*	[369,370]
Botryosphaeriaceae	*Barriopsis*	ITS	*tef1-α*	[371,372]
Didymellaceae	*Ascochyta*, *Boeremia*, *Didymella*, *Epicoccum*, *Phoma*	LSU and ITS	*rpb2*, *tub2* and *tef1-α*	[373,374,375,376]
Pleosporaceae	*Bipolaris*	*GPDH*	ITS, *tef1-α* and *GPDH*	[377]
Botryosphaeriaceae	*Botryosphaeria*	LSU, SSU and ITS	*tub* and *tef1-α*	[378,379]
Nectriaceae	*Calonectria*, *Cylindrocladium*	LSU and ITS	ITS, *tub*, *tef1-α*, *cmdA*, *His3* and *ACT*	[380,381,382,383,384]
Mycosphaerellaceae	*Cercospora*	LSU and ITS	ITS, *tef1-α*, *ACT*, *CAL*, *HIS*, *tub2*, *rpb2* and *GAPDH*	[385,386,387,388,389]
Cryptobasidiaceae	*Clinoconidium*	ITS and LSU	ITS and LSU	[390,391,392]
Choanephoraceae	*Choanephora*	ITS	ITS	[393]
Glomerellaceae	*Colletotrichum*	*GPDH*, *tub*; ApMat-Intergenic region of apn2 and MAT1-2-1 genes can resolve within the *C. gloeosporioides* complex	GS-glutamine synthetase-*CHS-1*, *HIS3*-Histone3 and *ACT*-Actin-Placement within the genus and also some species-level delineation	[394,395,396]
Schizoparmaceae	*Coniella*	LSU and ITS	ITS, LSU, *tef1-α*, *rpb2* and *His3*	[397,398,399,400,401]
Pleosporaceae	*Curvularia*	LSU	*GDPH*	[402,403,404]
Nectriaceae	*Cylindrocladiella*	ITS and LSU	*HIS*, *tef1-α* and *tub2*	[405,406]
Cyphellophoraceae	*Cyphellophora*	LSU and SSU	ITS, LSU, *tub2* and *rpb1*	[407,408]
Botryosphaeriaceae	*Diplodia*	ITS, *tef1-α* and *tub*	LSU and SSU	[378,409]
Botryosphaeriaceae	*Dothiorella*	*tub*	*tef1-α*	[378,410]
Elsinoaceae	*Elsinoe*	ITS	*rpb2* and *tef1-α*	[411,412]
Xylariaceae	*Entoleuca*	LSU and ITS	*rpb2* and *tub2*	[413]
Entylomataceae	*Entyloma*	ITS	ITS	[80,414,415]
Corticiaceae	*Erythricium*	LSU	ITS	[416]
Botryosphaeriaceae	*Eutiarosporella*	LSU and SSU	ITS and LSU	[372,417,418]
Hymenochaetaceae	*Fomitiporia*	ITS	LSU, ITS, *tef1-α* and *rpb2*	[419,420,421,422,423]
Hymenochataceae	*Fulvifomes*	LSU	ITS, *tef1-α* and *rpb2*	[424,425]
Nectriaceae	*Fusarium*	ATP citrate lyase (*Acl1*), *tef1-α* and ITS	Calmodulin encoding gene (*CmdA*), *tub2*, *tef1-α*, *rpb1* and *rpb2*	[426,427,428]
Ganodermataceae	*Ganoderma*	ITS	*rpb2* and *tef1-α*	[429,430,431,432,433,434,435]
Erysiphaceae	*Golovinomyces*	ITS and LSU	ITS and LSU, *IGS*, *rpb2* and *CHS*	[436,437,438,439,440]
Bondarzewiaceae	*Heterobasidion*	LSU	*rpb1* and *rpb2*	[441]
Nectriaceae	*Ilyonectria*	ITS, LSU, *tef1-α* and *tub2*	*tef1-α*, *tub2* and *His3*	[442,443,444,445,446]
Corticiaceae	*Laetisaria*, *Limonomyces*	LSU	ITS	[447,448]
Botryosphaeriaceae	*Lasiodiplodia*	SSU and LSU	ITS, *tef1-α* and *tub2*	[378,449]
Botryosphaeriaceae	*Macrophomina*	LSU and SSU	ITS, *tef1-α*, *ACT*, *CmdA* and *tub2*	[378,450]
Medeolariaceae	*Medeolaria*	ITS	ITS	[451]
Caloscyphaceae	*Caloscypha*	SSU and LSU	SSU, LSU	[452]
Meliolaceae	*Meliola*	LSU and SSU	ITS	[453,454]
Mucoraceae	*Mucor*	LSU and SSU	ITS and *rpb1*	[455,456,457,458,459]
Erysiphaceae	*Neoerysiphe*	ITS and LSU	ITS	[460,461,462]
Dermataceae	*Neofabraea*	LSU	ITS, LSU, *rpb2* and *tub2*	[463]
Botryosphaeriaceae	*Neofusicoccum*	SSU, LSU	ITS, *tef1-α*, *tub2* and *rpb2*	[464]
Nectriaceae	*Neonectria*	LSU, ITS, *tef1-α* and *tub2*	ITS, *tef1-α* and *tub2*	[446]
Sporocadaceae	*Neopestalotiopsis*	LSU	ITS, *tub2* and *tef1-α*	[465,466,467]
Didymellaceae	*Nothophoma*	LSU and ITS	*tub2* and *rpb2*	[468,469,470,471]
Sporocadaceae	*Pestalotiopsis*	LSU	ITS, *tub2* and *tef1-α*	[472,473]
Togninicaceae	*Phaeoacremonium*	SSU and LSU	*ACT* and *tub2*	[474,475,476]
Hymenochataceae	*Phellinotus*	LSU	ITS, *tef1-α* and *rpb2*	[477]
Hymenochaetaceae	*Phellinus*	LSU	ITS, *tef1-α* and *rpb2*	[478,479,480,481]
Phyllostictaceae	*Phyllosticta*	ITS	ITS, LSU, *tef1-α*, *GAPDH* and *ACT*	[57,482,483]
Peronosporacae	*Phytophthora*	LSU, SSU and *COX2*	LSU, *tub2* and *COX2*	[484,485]
Peronosporaceae	*Plasmopara*	LSU	LSU	[486]
Leptosphaeriaceae	*Plenodomus*	LSU	ITS, *tub2* and *rpb2*	[487]
Sporocadaceae	*Pseudopestalotiopsis*	LSU	ITS, *tub2* and *tef1*-α	[488,489]
Pyriculariaceae	*Pseudopyricularia*	LSU and *rpb1*	*ACT*, *rpb1*, ITS and *CAL*	[490,491]
Saccotheciaceae	*Pseudoseptoria*	LSU	LSU, ITS and *rpb2*	[492,493]
Rhizopodaceae	*Rhizopus*	ITS and *rpb1*	SSU, LSU and *ACT*	[494,495,496]
Xylariaceae	*Rosellinia*	LSU and ITS	ITS	[497,498,499,500]
Didymellaceae	*Stagonosporopsis*	ITS	*tub2* and *rpb2*	[373,501,502]
Pleosporaceae	*Stemphylium*	ITS	*CmdA* and *GAPDH*	[503,504,505,506]
Dothidotthiaceae	*Thyrostroma*	LSU	ITS, *tef1-α*, *rpb2* and *tub2*	[507,508]
Tilletiaceae	*Tilletia*	LSU	ITS	[509,510,511,512]
Ustilaginaceae	*Ustilago*	LSU	ITS	[53,513]
Venturiaceae	*Venturia*	LSU and SSU	ITS	[514,515]

## 6. Conclusions and Future Perspectives

After compiling this manuscript, it was concluded thatabout 4–5 million species of fungi are distributed all across the globe, and less than 2% of them have been described to date. Different estimates of fungal species ranging between 0.1–9.9 million have been provided by different mycologists working continuously on the taxonomy and diversity of fungi. The addition of new fungal taxa (genera and species) is an ongoing process, as a number of natural environments and a variety of habitatsare still waiting to be explored in terms of their fungal diversity. Based on a regression relationship between time and described fungal species, the description rate of fungi was calculated, and new proposed estimates were also presented. As per the description rate observed after this regression relationship, the estimation of 1.5 million fungal species could be achieved by the year 2184, while the estimation of 2.2 million could be achieved by 2210 and 5.1 million by 2245. 

Both classical and DNA-based methods to study fungi have their own utility and importance. While classical methods are still used widely due to low cost, ease of identifying species and ability to sample wide areas or many pieces of substrata, modern methods have also gained popularity due to their accuracy in characterizing the fungi which are not possible with traditional classical methods. When traditional morphology based species identification utilizes the overall morphology of an organism, DNA-based modern techniques require a very small amount of fungal sample. However, modern mycologists have accepted integrated approaches using both morphological and molecular data.

In the integral approach of traditional and modern methods of fungal analyses, fungal culture plays an important role. Production of different morphs on culture and other accessory structures are important for identification and characterization. Due to this non sporulation of many fungi neither on the natural substrate nor artificial culture media, the modern DNA-based technique proved to be more efficient to understand their taxonomy. New generation sequencing or metagenomic techniques are of much use to analyze the fungal diversity of different environments. There area large number of sequences from environmental samples (unculturable and dark taxa) available in GenBank which signifies the use of modern methods to describe many important fungi. The advancement in sequencing technologies of DNA and RNA is regularly helping researchers to study fungi in an integrative way and understand their biology, ecology and taxonomy in a better way. More than a billion HTS-derived ITS reads are available publicly in available databases and can be used by researchers during various mycological studies. It is important to use this data to assemble evidence hitherto overlooked, as well as new hypotheses, research questions and theories. If cultures of all fungi are deposited in culture collections and made easily available to researchers, it may perhaps add value to basic taxonomy research. 

The future of fungal taxonomy is challenging, as fungal systematics research requires well-trained mycologists with good expertise in traditional fungal classification, molecular systematics and bioinformatics/genomics. In order to produce experienced mycologists, the number of training programmers on fungal systematics should be organized more frequently for younger researchers. Molecular systematics training is comparatively expensive in nature and requires a decent facility for sequencing and/orcomputation. Research funding is not so uniform for taxonomic studies and is one of the possible reasons for declining fungal taxonomists. If this all goes at the same pace, the lack of well-trained fungal taxonomists will be a problem not only in the field of fungal taxonomy, but other scientific fields that rely on knowledge of fungal biodiversity and evolutionary biology. Therefore, adequate funding for research on taxonomic work is necessary to come out of this deprived situation. For young minds in college or plant pathology departments, more field research and highly advanced training programs should be organized to stimulate their interest in mycology.

## Figures and Tables

**Table 3:** An overview on DNA-based methods of fungal samples analyses.

Global Fungi Study ID	Substrate	Samples	Method	Sequencing Platform	ITS2 Sequences	Reference
Hartmann_2012_B1A3	–	6	Culture independent	454-pyrosequencing	2155088	[150]
Ihrmark_2012_3AE5	Soil, wood, wheat roots and hay	36	Culture independent	454-pyrosequencing	414896	[151]
Davey_2012_6F6A	Shoots of *Hylocomium splendens*, *Pleurozium schreberi*, and *Polytrichum commune*	301	Culture independent	454-pyrosequencing	296964	[152]
Peay_2013_74BB	Soil	36	Culture independent	454-pyrosequencing	86677	[153]
Davey_2013_7683	Shoots of *Dicranum scoparium*, *Hylocomium splendens*, *Pleurozium schreberi* and *Polytrichum commune*	454-pyrosequencing	Culture independent	454-pyrosequencing	313084	[154]
Talbot_2014_A187	Soil	555	Culture independent	454-pyrosequencing	16977	[155]
Tedersoo_2014_B9DD	Soil	360	Culture independent	454-pyrosequencing	1979803	[156]
Kadowaki_2014_B85B	Soil	46	Culture independent	454-pyrosequencing	66067	[157]
Geml_2014_2936	Soil	10	Culture independent	454-pyrosequencing	285031	[158]
Davey_2014_2252	Shoots of *Hylocomium splendens*	251	Culture independent	454-pyrosequencing	639746	[159]
McHugh_2015_CAE1	Soil	20	Culture independent	454-pyrosequencing	594424	[160]
DeBeeck_2014_14DC	Soil	20	Culture independent	454-pyrosequencing	32778	[161]
Yamamoto_2014_C3F7	Seedlings of *Quercus* sp.	431	Culture independent	454-pyrosequencing	59021	[162]
Walker_2014_22C1	Soil	24	Culture independent	454-pyrosequencing	34267	[163]
Veach_2015_7FDE	Soil	91	Culture independent	Illumina MiSeq	579967	[164]
Zhang_2015_A52F	Seven lichens speciesViz. *Cetrariella delisei*, *Cladonia borealis*, *C. arbuscula*, *C. pocillum*, *Flavocetraria nivalis*, *Ochrolechia frigida* and *Peltigera canina*	22	Culture independent	454-pyrosequencing	11087	[165]
Elliott_2015_7CC2	Soil	16	Culture independent	454-pyrosequencing	3896	[166]
Geml_2015_1A45	Soil	10	Culture independent	Ion Torrent	1098472	[167]
Hoppe_2015_BE27	Wood	48	Culture independent	454-pyrosequencing	121459	[168]
Jarvis_2015_B613	Roots of *Pinus sylvestris*	32	Culture independent	454-pyrosequencing	112333	[169]
Chaput_2015_41F7	Soil	4	Culture independent	Tag-encoded FLX amplicon pyrosequencing	1197	[170]
van_der_Wal_2015_1114	Sawdust from sapwood and heartwood of *Quercus robur*, *Rubus fruticosus*, *Sorbus aucuparia*, *Betula pendula*, *Pteridium aquilinum* and *Amelanchier lamarckii*	42	Culture independent	454-pyrosequencing	543801	[171]
Clemmensen_2015_B0AE	Soil	466	Culture independent	454-pyrosequencing GL FLX Titanium system	592836	[172]
Gao_2015_1CEF	Soil	24	Culture independent	454-pyrosequencing GL FLX Titanium system	93683	[173]
Liu_2015_6174	Soil	26	Culture independent	Roche FLX 454- pyrosequencing	53978	[174]
Oja_2015_88D4	*Cypripedium calceolus* (subfamily *Cypripedioideae*), *Neottia ovata* (*Epidendroideae*) and *Orchis militaris* (Orchidoideae) and Soil	158	Culture independent	454-pyrosequencing	63045	[175]
Goldmann_2015_EA26	Soil	48	Culture independent	454-pyrosequencer	140966	[176]
Tedersoo_2015_ED81	Soil	11	Culture independent	Illumina MiSeq	261751	[177]
Rime_2015_89DE	Soil	36	Culture independent	454-pyrosequencing GL FLX Titanium system	227118	[178]
Sterkenburg_2015_5E14	Soil	56	Culture independent	454-pyrosequencing	350560	[179]
Stursova_2016_D385	Soil	96	Culture independent	Illumina MiSeq	452546	[180]
Semenova_2016_576B	Soil	10	Culture independent	Ion Torrent sequencing	1007509	[181]
Santalahti_2016_74FC	Soil	117	Culture independent	454-pyrosequencing	739877	[182]
Rime_2016_E0E4	Soils and sediments	2	Culture independent	454-pyrosequencing	35937	[183]
RoyBolduc_2016_E50C	Root and soil	63	Culture independent	454-pyrosequencing	248325	[184]
RoyBolduc_2016_F11B	Soil	77	Culture independent	454-pyrosequencing	280272	[185]
Tedersoo_2016_TDEF	Soil	136	Culture independent	454-pyrosequencing	788372	[186]
UOBC_2016_5CA6	Soil	655	Culture independent	Illumina HiSeq	7138323	[187]
Urbina_2016_CE8E	Soil	21	Culture independent	Ion Torrent sequencing	564332	[188]
Valverde_2016_5E5C	Soil from the rhizosphere of *Welwitschia mirabilis*	8	Culture independent	454-pyrosequencing	2677	[189]
Nacke_2016_8F49	Soil from the rhizosphere *Fagus sylvatica* and *Picea abies*	160	Culture independent	454-pyrosequencing	386432	[190]
Newsham_2016_191B	Soil	29	Culture independent	454-pyrosequencing	509483	[191]
Nguyen_2016_D8E8	Shoots of *Picea abies*, *Abies alba*, *Fagus sylvatica*, *Acer pseudoplatanus*, *Fraxinus excelsior*, *Quercus robur*, *Pinus sylvestris*, *Betula pendula*, *Carpinus betulus* and *Quercus robur*	221	Culture independent	454-pyrosequencing	63853	[192]
Goldmann_2016_0757	Root and soil samples from beech-dominated plots	29	Culture independent	454-pyrosequencing	85867	[193]
Bahram_2016_7246	Soil	123	Culture independent	454-pyrosequencing	213249	[194]
Gehring_2016_E395	Roots and root-associated (rhizosphere) soil of sagebrush, cheatgrass, and rice grass plants	60	-	-	1161117	[195]
Gourmelon_2016_9281	Soil	32	Culture independent	Illumina MiSeq	91814	[196]
Bissett_AAAA_2016	Soil	2061	Culture independent	Illumina MiSeq	50810033	[197]
Cox_2016_EDC5	Soil	135	Culture independent	454-pyrosequencing	886200	[198]
Oh_2016_DEBA	Soil	12	Culture independent	454-pyrosequencing	98376	[199]
Frey_2016_5D5C	Soil	12	Culture independent	Illumina MiSeq v3	500999	[200]
Gannes_2016_5E98	Soil	23	Culture independent	Illumina MiSeq system	218946	[201]
Li_2016_1EBC	Soil	21	Culture independent	Illumina MiSeq system	129184	[202]
Kielak_2016_1110	Wood of *Pinus sylvestris*	75	Culture independent	454-pyrosequencing	1281356	[203]
Ji_2016_C06E	Soil	13	Culture independent	454-pyrosequencing	277	[204]
Baldrian_2016_DE02	Sawdust	118	Culture independent	llumina MiSeq	1205580	[205]
Barnes_2016_0042	Roots of *Cinchona calisaya*	21	Culture independent	llumina MiSeq	239387	[206]
Porter_2016_CD8D	Soil	2	Culture independent	454-pyrosequencing	20123	[207]
Zhou_2016_A8F1	Soil	126	Culture independent	Illumina MiSeq	3542416	[208]
Zhang_2016_1DA0	Soil	13	Culture independent	454-pyrosequencing	2362	[209]
Wang_2016_6223	Roots, stems, and sprouts of rice plant	1	Culture independent	Illumina MiSeq	1850	[210]
Zifcakova_2016_4C03	Soil	24	Culture independent	ILLUMINA HISEQ2000	123869	[211]
VanDerWal_2016_4C9C	Sawdust from sapwood and heartwood	130	Culture independent	454-pyrosequencing	1215932	[212]
Varenius_2017_BCFB	Soil	517	Culture independent	PacBio RSII platform by SciLifeLab	186474	[213]
van_der_Wal_2017_2D0D	Sawdust samples of *Larix* stumps, and *Quercus* stumps	88	Culture independent	Illumina MiSeq	877425	[214]
Wang_2017_7E18	Soil	6	Culture independent	454-pyrosequencing	53737	[215]
van_der_Wal_2017_3070	Soil	135	Culture independent	Illumina MiSeq	1572834	[216]
Vasutova_2017_3070	Soil	28	Culture independent	GS Junior sequencer	9370	[217]
Vaz_2017_C16E	Woody debris	2	Culture independent	Personal Genome Machine	11817	[218]
Yang_2017_2AFC	Soil	180	Culture independent	llumina MiSeq platform PE250	12688168	[219]
Wicaksono_2017_3B9E	Root samples of *Alnus acuminata*	24	Culture independent	Ion Torrent	3596531	[220]
Yang_2017_EB1D	Soil	26	Culture independent	Illumina MiSeq platform PE250	1450233	[221]
Zhang_2017_02C2	Plant litter and soil	54	Culture independent	Illumina MiSeq	2904476	[222]
Zhang_2017_F933	Peat soil	9	Culture independent	Illumina HiSeq2000	320199	[223]
Purahong_2017_8EFD	Wood sample	116	Culture independent	Genome Sequencer 454-FLX System	299831	[224]
Poosakkannu_2017_B342	Bulk soil, rhizosphere soil, and *D*. *flexuosa* Leaf	43	Culture independent	IonTorrent	259743	[225]
Bergottini_2017_02C2	Roots of *Ilex paraguariensis*	11	Culture independent	454-pyrosequencing	189048	[226]
Dean_2017_F5A5	Roots of *Glycine max* (soybean) and *Thlaspi arvense*	12	Culture independent	454-FLX titanium	12596	[227]
Fernandez_Martinez_2017_14C3	Soil	11	Culture independent	454-pyrosequencing	138524	[228]
Ge_2017_4DC8	Roots of *Quercus nigra*, *Q*. *virginiana*, *Q*. *laevis*, *Carya* cf. *glabra*, *Carya* cf. *tomentosa* as well as several *Carya* and *Quercus* spp.	9	Culture independent	454-pyrosequencing	44	[229]
Gomes_2017_2AFC	Roots of *Thismia* sp.	61	Culture independent	Ion Torrent	4067438	[230]
Almario_2017_2082	Root and rhizosphere of *Arabis alpina*	26	Culture independent	Illumina Miseq	805679	[231]
Anthony_2017_647F	Soil	142	Culture independent	Illumina Miseq	12453259	[232]
Grau_2017_E29A	Soil	27	Culture independent	Ion Torrent	960177	[233]
Hiiesalu_2017_E29A	Soil	1	Culture independent	454-pyrosequencing	4616	[234]
Nguyen_2017_6F2C	Leaf samples of *Betula pendula*	20	Culture independent	454-pyrosequencing	1318	[235]
Kolarikova_2017_EB1D	Roots of *Salix caprea* and *Betula pendula*	24	Culture independent	454-pyrosequencing	47543	[236]
Kyaschenko_2017_89D4	Soil	30	Culture independent	PacBio sequencing	64010	[237]
Oja_2017_AD29	Roots and rhizosphere soil of	333	Culture independent	454-pyrosequencing	446296	[238]
Miura_2017_2BE5	Leaves and berries of grapes	36	Culture independent	Illumina MiSeq	2250530	[239]
Oono_2017_B342	Needles of *Pinus taeda*	143	Culture independent	Illumina MiSeq	9755183	[240]
Kamutando_2017_6F2C	Soil	3	Culture independent	Illumina MiSeq	4	[241]
Shen_2017_C7F4	Soil	1	Culture independent	Illumina MiSeq	1	[242]
Smith_2017_2AFC	Root of *Dicymbe corymbosa*	8	Culture independent	454-pyrosequencing	94	[243]
Tian_2017_F933	Soil	3	Culture independent	454-GS FLX+pyrosequencing machine	25001	[244]
Tu_2017_BCFB	Soil	60	Culture independent	Illumina MiSeq	696557	[245]
Sharma_Poudyal_2017_F933	Soil	53	Culture independent	454-FLX titanium	7680	[246]
Cross_2017_2AFC	Leaflet, petiole upper and petiole base tissues of ash leaves of *Fraxinus excelsior* (common ash)	27	Culture independent	454-pyrosequencing	171094	[247]
Kazartsev_2018_1115	Bark of *Picea abies*	20	Culture independent	454-pyrosequencing	22918	[248]
Bickford_2018_2EE0	Roots of *Phragmites* spp.	3	Culture independent	PacBio-RS II	66439	[249]
Cline_2018_0BCC	Wood of *Betula papyrifera*	15	Culture independent	454-FLX titanium	660	[250]
Cregger_2018_added	Roots, stems, and leaves of *Populus deltoides* and the *Populus trichocarpa* × *deltoides* hybrid	290	Culture independent	Illumina MiSeq	14767409	[251]
Marasco_2018_DBE1	Rhizosheath-root system of *Stipagrostis sabulicola*, *S. seelyae* and *Cladoraphis spinosa*	49	Culture independent	Illumina MiSeq	4694085	[252]
Glynou_2018_445A	Roots of nonmycorrhizal *Microthlaspi* spp.	5	Culture independent	Illumina Miseq	7	[253]
Montagna_2018_E316	Soil	24	Culture independent	Illumina Miseq	2475767	[254]
Schlegel_2018_A231	Leaves of *Fraxinus* spp. and *Acer pseudoplatanus*	353	Culture independent	Illumina MiSeq	24198214	[255]
SchneiderMaunoury_2018_51AB	Different plant species	78	Culture independent	Ion Torrent	352332	[256]
Schon_2018_01F4	Soil	18	Culture independent	Illumina MiSeq	235709	[257]
Rasmussen_2018_C8E6	Root samples	228	Culture independent	Illumina MiSeq	428044	[258]
Rogers_2018_147F	Hemlock stems	6	Culture independent	Illumina MiSeq	675067	[259]
Purahong_2018_14C0	Deadwood logs	297	Culture independent	454-pyrosequencing	2034928	[260]
Qian_2018_2B1E	Leaves of *Mussaenda shikokiana*	20	Culture independent	Illumina MiSeq	449179	[261]
Park_2018_569C	*Calanthe* species: *C*. *aristulifera*, *C*. *bicolor*, *C*. *discolor*, *C*. *insularis* and *C*. *striata*	12	Culture independent	454-GS FLX +System	65867	[262]
Mirmajlessi_2018_765D	Soil	40	Culture independent	Illumina MiSeq	1077125	[263]
Purahong_2018_9F2E	Wood samples	96	Culture independent	454-pyrosequencing	656682	[264]
Si_2018_53B6	Soil	27	Culture independent	Illumina MiSeq	692169	[265]
Saitta_2018_51C8	Soil	16	Culture independent	Illumina MiSeq	4923667	[266]
Santalahti_2018_3794	Soil	38	Culture independent	454-pyrosequencing	218387	[267]
Sukdeo_2018_1DF4	Soil	126	Culture independent	Illumina MiSeq	32336646	[268]
Zhu_2018_1E38	Soil	12	Culture independent	Illumina MiSeq	1031479	[269]
Zhang_2018_F81F	Soil	106	Culture independent	Illumina HiSeq	1673070	[270]
Zhang_2018_491A	Bare sand, algal crusts, lichen crusts, and moss crusts	17	Culture independent	Illumina Miseq	442056	[271]
Sun_2018_1B01	Soil	36	Culture independent	Illumina Miseq	1188520	[272]
Weissbecker_2019_6A75	Soil	394	Culture independent	GS-FLX + 454 pyrosequencer	1109208	[273]
Purahong_AD_2019	Wood chips of rotted heartwood deadwood from *C*. *carlesii*	3	Culture independent	PacBio RS II system	22886	[274]
Egidi_AD_2019	Soil	161	Culture independent	Illumina MiSeq	14131987	[275]
Froeslev_2019_CA74	Soil	276	Culture independent	Illumina MiSeq	6114124	[276]
Ogwu_2019_38FE	Soil	13	Culture independent	Illumina Miseq	724483	[277]
Ovaskainen_2019_air	Soil particles, spores, pollen, bacteria, and small insects	75	Culture independent	Illumina Miseq	935812	[278]
Qian_2019_9691	Leaves and soil	30	Culture independent	Illumina HiSeq	2133292	[279]
Ramirez_2019_D0B2	Soil	810	Culture independent	Illumina Miseq	6555903	[280]
Pellitier_2019_82BC	Bark of black oak (*Quercus velutina*), white oak (*Q. alba*), red pine (*Pinus resinosa*), eastern white pine (*P. strobus*) and red maple (*Acer rubrum*)	15	Culture independent	Illumina MiSeq	10649956	[281]
Semenova-Nelsen_2019_add	Litter and the uppermost soil	121	Culture independent	Illumina MiSeq	3205748	[282]
Sheng_2019_66AC	Soil	16	Culture independent	Illumina MiSeq	447840	[283]
Shigyo_2019_5B19	Soil	144	Culture independent	Illumina MiSeq	4353704	[284]
Schroter_2019_1B64	Fine roots and soil	3	Culture independent	Roche GS-FLX+ pyrosequencer	144	[285]
Singh_2019_EA7F	Fine roots and soil	96	Culture independent	Illumina MiSeq	3138303	[286]
Song_2019_ad2	Soil	46	Culture independent	Illumina MiSeq	920391	[287]
U’Ren_2019_add	Fresh, photosynthetic tissues of a diverse range of plants and lichens	486	Culture-based sampling and culture-independent	Illumina MiSeq	5671834	[288]
Unuk_2019_567A	Fine roots and soil	30	Culture independent	Ilumina MiSeq	470786	[289]
Araya_2019_add	Soil	36	Culture independent	Illumina MiSeq	8083471	[290]
Alvarez-Garrido_2019_add	Root tips from *A*. *pinsapo* trees following the trunk to the superficial secondary roots	76	Culture independent	Illumina MiSeq	1795423	[291]
Wei_2019_3796	Soil	1	Culture independent	Illumina HiSeq	18	[292]
Pan_2020_addZ	Soil from the rhizosphere of potato	1	Culture independent	Illumina MiSeq	2	[293]
Detheridge_2020_Z	Soil	70	Culture independent		1832454	[294]
Li_2020_AS	Soil	19	Culture independent	Illumina MiSeq	116660	[295]

**Table 4:** An overview on culture dependentand culture independent analyses of fungal samples with respect to location, source, sequencing, observation method and target gene.

Location	Source	Sequencing	Method			Target Gene	Reference
Woods Hole Harbor Massachusetts	Wood	–	Culture dependent	Direct observation	–	–	[296]
Atlantic Ocean	Water	–	Culture dependent	Incubation of sample and direct observation	–	–	[297]
Rumanian coast of the Black Sea	Calcareous substances	–	Culture dependent	Incubation of sample and direct observation	–	–	[298]
Iceland-Faroe ridge	Water	–	Culture dependent	Incubation of sample and direct observation	–	–	[299]
Bahamas	Wood	–	Culture dependent	Incubation of sample and direct observation	–	–	[300]
Bay of Bengal and Arabian Sea	Sediment		Culture dependent	Culture media	–	–	[301]
Northwest Pacific Ocean (Sagami Bay and Suruga Bay; Palau-Yap Trench and Mariana Trench)	Sediments	Sanger	Culture dependent	Culture media	–	ITS and 5.8S	[302]
Guaymas Basin hydrothermal vent	Sediment	Sanger	Culture independent	–	Clone library	SSU	[303]
Mid-Atlantic Ridge hydrothermal area	Sediment	Sanger	Culture independent	–	Clone library	SSU	[304]
Chagos Trench, Indian Ocean	Sediment	–	Culture dependent/Direct detection	Culture media	–	–	[305]
Peru Margin	Sediment	Sanger	Culture dependent	Culture media	–	SSU	[306]
Central Indian Basin	Sediment	–	Culture dependent	Culture media	–	–	[307]
Kuroshima Knoll in Okinawa	Sediment	Sanger	Culture dependent		Clone library	SSU	[308]
Central Indian Basin	Sediment	Sanger	Culture dependent	Culture media	–	–	[309]
Different locations	Water and sediment	Sanger	Culture dependent		Clone library	SSU	[310]
South China Sea	Sediment	Sanger	Culture dependent	–	Clone library	ITS	[311]
Lost City	Water	Sanger	Culture dependent	–	Clone library	SSU	[312]
Central Indian Basin	Sediment		Direct detection	–	–	–	[313]
Vailulu’u is an active submarine volcano at the eastern end of the Samoan volcanic chain	Water	Sanger	Culture dependent	Culture media	–	ITS	[314]
Vanuatu archipelago	Deepsea water, wood and debris	Sanger	Culture dependent	Culture media	–	SSU and LSU	[315]
East Pacific Rise, Mid-Atlantic Ridge and Lucky Strike	Deepsea hydrothermal ecosystem	Sanger	Culture dependent/Cultureindependent	Culture media	Clone library	SSU	[316]
Southwest Pacific	Deepsea hydrothermal ecosystems	Sanger	Culture dependent	Culture media	–	SSU	[317]
Different locations	Deep-sea hydrothermal ecosystems	Sanger	Culture dependent	Culture media	–	LSU	[318]
Japanese islands, including a sample from the deepest ocean depth, the Mariana Trench	Sediment	Sanger	Culture independent	–	Clone library	SSU, ITS and LSU	[319]
Southern East Pacific Rise	Water and bivalves	Sanger	Culture independent	–	Clone library	SSU	[320]
Central Indian Basin	Sediment	Sanger	Culture dependent	Culture media		Full ITS and SSU	[321]
Southern Indian Ocean	Sediment	Sanger	Culture independent	–	Clone library	SSU	[322]
Peru Margin and the Peru Trench	Sediment	Sanger	Culture independent	–	Clone library	SSU	[323]
Puerto Rico Trench	Water	Sanger	Culture independent	–	Clone library	SSU	[324]
Sagami-Bay	Deep-sea methane cold-seep sediments	Sanger	Culture independent	–	Clone library	SSU	[325]
Marmara Sea	Sediment	Sanger and 454-pyrosequencing	Culture independent	–	Clone library	SSU	[326]
Central Indian Basin - Several stations	Sediment	Sanger	Culture independent	–	Clone library	Full ITS and SSU	[327]
Central Indian Basin - Several stations	Sediment	Sanger	Culture dependent/Culture independent	Culture media	Clone library	SSU (Fungal isolates)/ITS (DNA sediment)	[328]
Central Indian Basin - Several stations	Sediment	Sanger	Culture independent cloning	–	Clone library	Full ITS and SSU	[328]
Alaminos Canyon 601 methane seep in the Gulf of Mexico	Methane seeps sediment	Sanger	Culture independent	–	Clone library	ITS and LSU	[329]
The area surrounding the DWH oil spill in the Gulf of Mexico	Deep-sea samples from the area surrounding the Deepwater Horizon oil spill	454-pyrosequencing	Culture independent	–	Shotgun	*assA* and *bssA*	[330]
Hydrate Ridge, Peru Margin, Eastern Equatorial Pacific	Sediment	Sanger and 454-pyrosequencing	Culture independent	–	TRFLP/Metatranscriptomics	SSU	[331]
Peru Margin	Sediment	Illumina	Culture independent		Metatranscriptomics	–	[331]
South China Sea	Sediment	Sanger	Culture dependent	Culture media	–	Full ITS	[332]
Mediterranean Sea	Hypsersaline anoxic basin	454-pyrosequencing	Culture independent	–	–	SSU	[333]
Canterbury basin, on the eastern margin of the South Island of New Zealand	Sediment Ocean Drilling Program	454-pyrosequencing	Culture independent	–	Metatranscriptomics	ITS and SSU	[334]
The Pacific Ocean and MarianaTrench	Sediment	Sanger	Culture independent	–	Clone library	ITS	[335]
East Indian Ocean	Sediment	Sanger	Culture dependent/Culture independent	Culture media	Clone library	ITS	[336]
Canterbury basin, on the eastern margin of the South Island of New Zealand	Sediment	Sanger	Culture dependent	Culture media	–	SSU, ITS and LSU	[337]
Urania, Discovery and L’Atalante basins	Hypsersaline anoxic basin	Illumina	Culture independent	–	Metatranscriptomics	–	[338]
Several locations around the world/The ICoMM data set	Pelagic and benthic samples	454-pyrosequencing	Culture independent	–	–	SSU	[339]
The Pacific Ocean and MarianaTrench	Sediment	Sanger	Culture independent	–	Clone library	ITS, SSU and LSU	[340]
Okinawa	Sediment	Illumina	Culture independent	–	–	ITS	[341]
Southwest Indian Ridge (SWIR)	Sediment and Deepsea hydrothermal ecosystems	Sanger and Illumina	Culture dependent/Culture independent	With and without Culture media	–	ITS	[342]
Continental margin of Peru	Sediment	Illumina	Culture independent	–	–	SSU	[343]
North Atlantic and Arctic Basin	Marine snow		Culture independent	–	CARD-FISH	–	[344]
Northern Chile	Water	Sanger	Culture dependent	–	–	Full ITS	[345]
The Sao Paulo Plateau	Asphalt seeps	Ion Torrent	Culture independent	–	–	ITS	[346]
Peru Margin	Sediment	Illumina	Culture independent	–	Metatranscriptomics		[347]
East Pacific	Sediment	Sanger	Culturedependent	Culture media		Full ITS	[348]
The Ionian Sea (Central Mediterranean Sea)	Sediment	Illumina	Culture independent	–	FISH	ITS	[349]
South-central western Pacific Ocean	Water	Illumina	Culture independent	–	–	SSU	[350]
Challenger deep	Water	Illumina	Culture independent	–	–	ITS	[351]
Mexican Exclusive Economic Zone-Gulf of Mexico	Sediment	Illumina	Culture independent	–	–	ITS	[352]
Yap Trench	Sediment	Sanger and Illumina	Culture dependent/Culture independent	–	–	ITS	[353]
Mexican Exclusive Economic Zone-Gulf of Mexico	Sediment	Sanger	Culture dependent	Culture media	–	Full ITS and *tub*	[354]

**Table 5:** Databases and tools for sequence-based classification and identification.

General Identification Tools and Data Repositories	
BOLD	http://www.boldsystems.org/ (accessed on 6 November 2021)
Westerdijk Fungal BiodiversityInstitute	https://wi.knaw.nl/page/Collection (accessed on 6 November 2021)
CIPRES	https://www.phylo.org/ (accessed on 6 November 2021)
Dryad	http://datadryad.org/ (accessed on 6 November 2021)
FUSARIUM-ID	http://isolate.fusariumdb.org/ (accessed on 6 November 2021)
One Stop Shop Fungi	http://onestopshopfungi.org/ (accessed on 6 November 2021)
GreenGenes	http://greengenes.lbl.gov/cgi-bin/nph-index.cgi (accessed on 6 November 2021)
MaarjAM	http://maarjam.botany.ut.ee/ (accessed on 6 November 2021)
Mothur	http://www.mothur.org/ (accessed on 6 November 2021)
Naïve Bayesian Classifier	http://aem.asm.org/content/73/16/5261.short?rss=1&ssource=mfc (accessed on 6 November 2021)
Open Tree of Life	http://www.opentreeoflife.org/ QIIME http://qiime.org/ (accessed on 6 November 2021)
PHYMYCO database	http://phymycodb.genouest.org/ (accessed on 6 November 2021)
RefSeq Targeted Loci	http://www.ncbi.nlm.nih.gov/refseq/targetedloci/ (accessed on 6 November 2021)
Ribosomal Database Project (RDP)	http://rdp.cme.msu.edu/ (accessed on 6 November 2021)
Silva	http://www.arb-silva.de/ (accessed on 6 November 2021)
TreeBASE	http://treebase.org/ (accessed on 6 November 2021)
TrichoBLAST	http://www.isth.info/tools/blast/ (accessed on 6 November 2021)
UNITE	http://unite.ut.ee/ (accessed on 6 November 2021)
United Kingdom National Culture Collection	http://www.ukncc.co.uk/ (accessed on 6 November 2021)
**Data standards**	
BIOM	http://biom-format.org/ (accessed on 6 November 2021)
MIMARKS	http://www.nature.com/nbt/journal/v29/n5/full/nbt/1823.html (accessed on 6 November 2021)
Darwin	Core http://rs.tdwg.org/dwc/ (accessed on 6 November 2021)
**Genomics databases and tools**	
AFTOL	http://aftol.umn.edu/ (accessed on 6 November 2021)
1000 Fungal Genomes Project (1KFG)	http://1000.fungalgenomes.org/home/ (accessed on 6 November 2021)
FungiDB	http://fungidb.org/fungidb/ (accessed on 6 November 2021)
GEBA	http://jgi.doe.gov/our-science/science-programs/microbial-genomics/phylogenetic-diversity/ (accessed on 6 November 2021)
MycoCosm	http://genome.jgi.doe.gov/programs/fungi/index.jsf (accessed on 6 November 2021)
**Functional database**	
FUNGuild	http://github.com/UMNFuN/FUNGuild (accessed on 6 November 2021)
**Nomenclature and nomenclatural databases and organizations**	
Catalogue of Life (COL)	http://www.catalogueoflife.org/ (accessed on 6 November 2021)
EPPO-Q-bank	http://qbank.eppo.int/ (accessed on 6 November 2021)
Faces of Fungi	http://www.facesoffungi.org/ (accessed on 6 November 2021)
Index Fungorum	http://www.indexfungorum.org/ (accessed on 6 November 2021)
International code of nomenclature for algae, fungi, and plants (ICNAFP)	http://www.iapt-taxon.org/nomen/main.php (accessed on 6 November 2021)
International Commission on the Taxonomy of Fungi (ICTF)	http://www.fungaltaxonomy.org/ (accessed on 6 November 2021)
List of prokaryotic names with standing in nomenclature (LPSN)	http://www.bacterio.net/ (accessed on 6 November 2021)
MycoBank	http://www.mycobank.org/ (accessed on 6 November 2021)
Outline of fungi	http://www.outlineoffungi.org/ (accessed on 6 November 2021)
**Biodiversity collections databases**	
Global Biodiversity Information Facility (GBIF)	http://www.gbif.org/ (accessed on 6 November 2021)
iDigBio	http://www.idigbio.org/ (accessed on 6 November 2021)
MycoPortal	http://mycoportal.org/portal/index.php (accessed on 6 November 2021)
World Federation of Culture Collections (WFCC)	http://www.wfcc.info/ (accessed on 6 November 2021)

**Table 6:** Sequence Independent methods and High-throughput sequencing platforms.

Sequencing Independent Methods	High-Throughput Sequencing Platforms
ARDRA (Amplified Ribosomal DNA Restriction Analysis)	454 Pyrosequencing (second-generation platform)
ARISA (Amplified Intergeneric Spacer Analysis)	Illumina MiSeq sequencing (second-generation)
DGGE (Denaturing Gradient Gel Electrophoresis)	Ion Torrent PGM and GeneStudio
FISH (Fluorescence in Situ Hybridization)	PacBio RSII and Sequel (This third-generation HTS platform)
LAMP (Loop-Mediated Isothermal Amplification)	Oxford Nanopore MinION, GridION and PrometION (third-generation)
MT-PCR (Multiplexed tandem PCR)	–
RCA (Rolling Circle Amplification)	–
RDBH (Reverse Dot Blot Hybridization)	–
RFLP (Restriction Fragment Length Polymorphism)	–
SSCP (Single-Strand Conformation Polymorphism)	–
TGGE (Thermal Gradient Gel Electrophoresis)	–
TRFLP (Terminal Restriction Fragment Length Polymorphism)	–

## Data Availability

Not applicable.
